# Is eucalyptus oil an effective antifungal treatment for onychomycosis with and without nail matrix infection?

**DOI:** 10.1186/1757-1146-8-S2-P1

**Published:** 2015-09-22

**Authors:** Cassandra Bramston, Caroline Robinson

**Affiliations:** 1 Podiatry Department, Charles Sturt University, Albury, NSW, 2640, Australia

**Keywords:** Eucalyptus oil, onychomycosis, dermatophyte moulds, fungal nail infection

## Background

Onychomycosis is a fungal infection causing progressive destruction of the nail elements, with age increasing frequency of infection and potential for complications. The condition accounts for almost half of all nail issues yet continues to be largely under-reported and untreated. This study will investigate the antifungal efficacy of eucalyptus oil *in vivo*, for fungal nail infections with and without nail matrix infection.

## Methods

A longitudinal prospective study was used to monitor the changes in toenail mycotic infections over a four-month period, using undiluted eucalyptus oil as a topical antifungal agent. Data were collected from 22 participants (14 men and 8 women) aged between 40 and 84 with a total of 70 toenails, 35 with nail matrix infection and 35 without. To review the effect of eucalyptus oil on the clinical appearance of the fungal infected nail plates, toenails were monitored at four-weekly intervals for a period of four months. The participants’ satisfaction with the therapy was assessed using the OnyCOE-t questionnaire.

## Results

Of the 70 nails analysed, the patterns of infection were classified as proximal subungual onychomycosis (49%), distal lateral subungual onychomycosis (47%), white superficial onychomycosis (3%), and total dystrophic (1%). Almost half of all participants (45%) had onychomycosis affecting only one toenail. Only 23% of participants had previously treated the infection and the mean duration of infection was 10 years and 3 months.

Nails with superficial onychomycosis (n=2) were found to have 86% clearance of infection after four months. One third of all nails (n=11) with distal lateral subungual onychomycosis and 50% (n=17) of nails with proximal subungual onychomycosis demonstrated a zone of clearance at the proximal nail plate tissue, suggesting a fungistatic effect of the eucalyptus oil.

## Conclusions

Topical eucalyptus oil is more effective as an antifungal treatment for fungal infected toenails without nail matrix infection. Eucalyptus oil may provide an acceptable and cheaper alternative to prescription topical antifungal agents, for people with white superficial onychomycosis or distal lateral subungual fungal nail infections.

